# Anisotropic Bi-Layer Hydrogel Actuator with pH-Responsive Color-Changing and Photothermal-Responsive Shape-Changing Bi-Functional Synergy

**DOI:** 10.3390/gels9060438

**Published:** 2023-05-25

**Authors:** Chao Ma, Shuyi Peng, Lian Chen, Xingyu Cao, Ye Sun, Lin Chen, Lang Yang, Chunming Ma, Qijie Liu, Zhenzhong Liu, Shaohua Jiang

**Affiliations:** 1 State Key Laboratory of Marine Resource Utilization in South China Sea, Hainan University, Haikou 570228, China; 20080500210019@hainanu.edu.cn (C.M.); 20080500210021@hainanu.edu.cn (S.P.); 20085600210002@hainanu.edu.cn (X.C.); sunye@hainanu.edu.cn (Y.S.); 22110805000014@hainanu.edu.cn (L.C.); yl13339833255@163.com (L.Y.); 2 Jiangsu Co-Innovation Center of Efficient Processing and Utilization of Forest Resources, International Innovation Center for Forest Chemicals and Materials, College of Materials Science and Engineering, Nanjing Forestry University, Nanjing 210037, China; lianchen@njfu.edu.cn; 3 Shenzhen Institute of Advanced Electronic Materials—Shenzhen Fundamental Research Institutions, Shenzhen Institutes of Advanced Technology, Chinese Academy of Sciences, Shenzhen 518055, China; 4 Taizhou Key Laboratory of Medical Devices and Advanced Materials, Research Institute of Zhejiang University, Taizhou 318000, China; tanksman@163.com (Q.L.); zzliu@zju.edu.cn (Z.L.)

**Keywords:** intelligent hydrogels, stimuli-responsive shape change, stimuli-responsive color change, bi-functional synergy, biomimetics

## Abstract

Stimuli-responsive color-changing and shape-changing hydrogels are promising intelligent materials for visual detections and bio-inspired actuations, respectively. However, it is still an early stage to integrate the color-changing performance and shape-changing performance together to provide bi-functional synergistic biomimetic devices, which are difficult to design but will greatly expand further applications of intelligent hydrogels. Herein, we present an anisotropic bi-layer hydrogel by combining a pH-responsive rhodamine-B (RhB)-functionalized fluorescent hydrogel layer and a photothermal-responsive shape-changing melanin-added poly (N-isopropylacrylamide) (PNIPAM) hydrogel layer with fluorescent color-changing and shape-changing bi-functional synergy. This bi-layer hydrogel can obtain fast and complex actuations under irradiation with 808 nm near-infrared (NIR) light due to both the melanin-composited PNIPAM hydrogel with high efficiency of photothermal conversion and the anisotropic structure of this bi-hydrogel. Furthermore, the RhB-functionalized fluorescent hydrogel layer can provide rapid pH-responsive fluorescent color change, which can be integrated with NIR-responsive shape change to achieve bi-functional synergy. As a result, this bi-layer hydrogel can be designed using various biomimetic devices, which can show the actuating process in the dark for real-time tracking and even mimetic starfish to synchronously change both the color and shape. This work provides a new bi-layer hydrogel biomimetic actuator with color-changing and shape-changing bi-functional synergy, which will inspire new strategies for other intelligent composite materials and high-level biomimetic devices.

## 1. Introduction

Stimuli-responsive shape-changing hydrogels and stimuli-responsive color-changing hydrogels are two of the most important intelligent hydrogels [1,2], which have been greatly developed over the past two decades and are both promising cutting-edge smart materials for widely high value-added applications [3,4,5,6].

Stimuli-responsive color-changing hydrogels can change the intensity and wavelength of their color in response to various external signals [1]. Nowadays, many color-changing hydrogels have been developed, which can provide good detection in diverse fields, such as heavy metal sensing, biomedicine, intelligent displaying, and so on. There are several main kinds of color-changing hydrogels based on different mechanisms, including (1) traditional organic molecules/polymers with fluorescent groups with aggregation-caused quenching (ACQ) effect [7,8,9]; (2) recent organic molecules/polymers with aggregation-induced emission (AIE) effect [10,11,12,13,14,15]; (3) various luminescent nanoparticles (such as fluorescent carbon dots [16,17,18], photonic crystals [19,20,21], and quantum dots [22,23,24]).

At the same time, stimuli-responsive shape-changing hydrogels have also been rapidly developed, which can respond to many kinds of external stimuli, including pH, ionic strength, temperature, chemical/biochemical molecules, electric/magnetic fields, etc. [25,26,27,28,29,30,31]. Furthermore, bio-inspired natural anisotropic structures and various anisotropic structures of hydrogels have been explored to design stimuli-responsive complex shape changes [32,33,34,35]. For instance, layered structures [36,37], gradient structures [38,39], oriented structures [40,41,42], pattern structures [43,44,45,46], and programmable 4D-printing anisotropic structures [47,48,49] have been obtained, which can provide various complex shape changes, including 1D to 2D bending and folding, 1D/2D to 3D curling and torsion, and 3D to 3D buckling, swing, and spiral. In addition, the introduction of intelligent response materials and the size design of materials based on various design processes further expand the types of stimulus responses and response speed. It should be noted that in recent years, there has also been a new trend of combining two/more techniques to design more complex and controllable anisotropic structures. In addition, most recently, remotely controlled shape-changing hydrogels have been explored in response to magnetic [38], electric [50], and light [42] stimuli, which can provide ultrafast and precisely programmable actuations. These anisotropic stimuli-responsive shape-changing hydrogels have been widely utilized in soft micro-actuators/robots, drug-controlled release, intelligent valves, and many other emerging fields.

However, most intelligent hydrogels can only have one intelligent function and cannot reach a high-level biomimetic bi/multi-functional synergistic effect. Looking back on nature, many creatures (such as starfish, octopuses, chameleons, etc.) have the ability to change color and shape. Furthermore, these creatures can use these two functions together when needed to better capture prey, protect themselves, and transmit signals. Although a few scientists have successfully explored intelligent hydrogels to achieve color-changing and shape-changing bi-functional synergy [51,52,53,54], it is still an early stage to integrate the two intelligent functions together to provide bi-functional synergistic biomimetic devices, which are severely difficult to design but will greatly expand the applications of intelligent hydrogels.

**Scheme 1:**
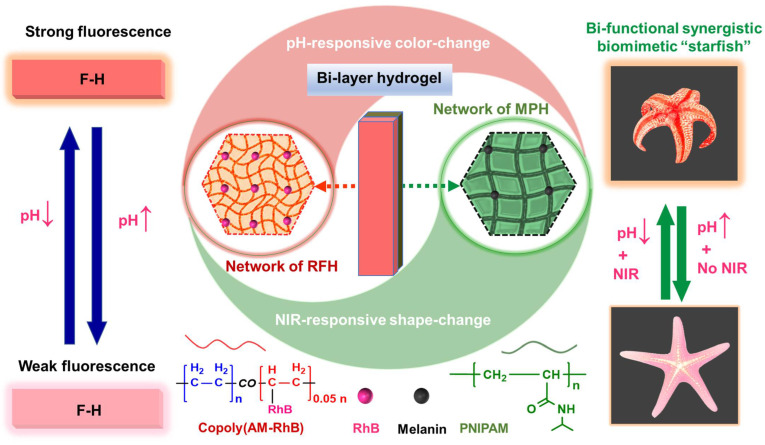
Schematic illustration of the composite actuator structure and fluorescent color-changing and shape-changing bi-functional synergistic biomimetic performance.

Herein, we have explored an anisotropic bi-layer hydrogel by combining a pH-responsive rhodamine-B (RhB) functionalized fluorescent hydrogel (RFH) layer and a photothermal-responsive shape-changing melanin-added poly (N-isopropylacrylamide) (PNIPAM) hydrogel (MPH) layer with fluorescent color-changing and shape-changing bi-functional synergy (Scheme 1). This bi-layer hydrogel can obtain fast and complex actuations under 808 nm irradiation of near-infrared (NIR) light due to both the melanin-composited temperature-responsive PNIPAM hydrogel with high efficiency of photothermal conversion and the anisotropic structure of this bi-hydrogel. Furthermore, the RhB-functionalized fluorescent hydrogel layer can provide rapid pH-responsive fluorescent color change, which can be integrated with NIR-responsive shape change to achieve bi-functional synergy. Therefore, this bi-layer hydrogel can be designed using various biomimetic devices, which can show the actuating process in the dark for real-time tracking and even mimetic starfish to change color and shape synchronously. This work will provide a new bi-layer hydrogel biomimetic actuator with color-changing and shape-changing bi-functional synergy for high-level biomimetic devices.

## 2. Results and Discussion

### 2.1. Fabrication of the Bi-Layer Anisotropic Hydrogel

**Figure 1:**
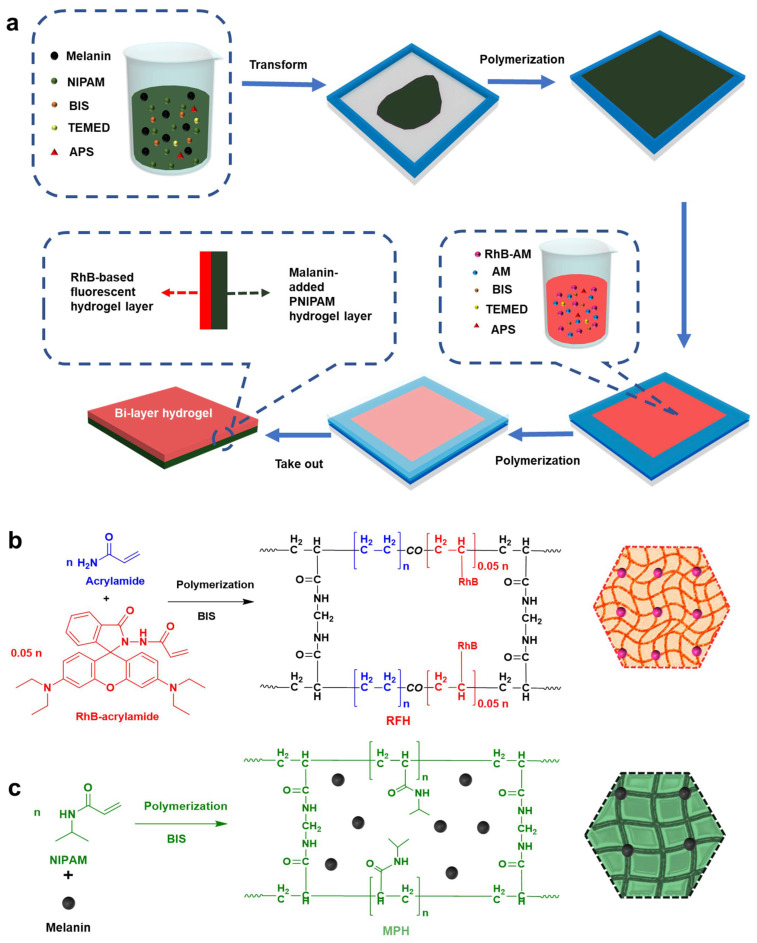
(**a**) The preparation diagram of the bi-layer hydrogel. (**b**) The chemical reaction and the illustration of the chemical structures of the RFH layers. (**c**) The chemical reaction and the illustration of the chemical structures of the MPH layers.

The fabrication process and the chemical structure of this bi-layer anisotropic hydrogel are shown in Figure 1. The bi-layer hydrogel can be prepared simply by hydrogelating the second RhB-functionalized fluorescent polyacrylamide (PAM) hydrogel layer on the first melanin-added PNIPAM hydrogel layer via polymerization at room temperature (Figure 1a). Melanin is a natural molecule with high-efficiency photothermal conversion, which can provide excellent photothermal-responsive shape-changing performance to the melanin-added PNIPAM hydrogel layer. In addition, the two hydrogel layers can form a bi-layer anisotropic structure with complex shape-changing properties. Furthermore, this bi-layer hydrogel can provide both pH-responsive fluorescent color change and photothermal complex shape change, which can provide bi-functional synergistic effects. The synthesis and preparation of pH-responsive rhodamine B (RhB) are shown in Figure S1. The chemical reaction and chemically crosslinked networks of the RhB-functionalized fluorescent hydrogel (RFH)/melanin-added PNIPAM hydrogel (MPH) bi-layer hydrogel are shown in Figure 1b and Figure 1c, respectively, which manifested their pH-responsiveness and photothermal-responsiveness. Last but not least, the chemical structures of the two hydrogel layers also indicated that they can be bonded to each other due to the monomer diffusion and the subsequent interpenetration of the two crosslinked networks between the interface.

**Figure 2:**
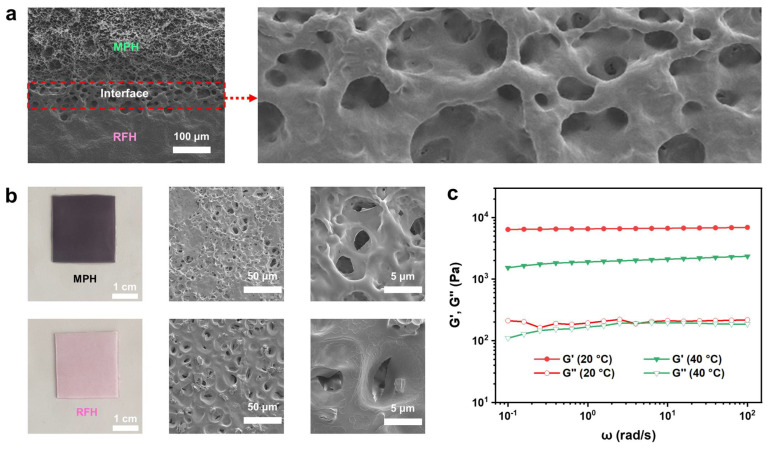
(**a**) The SEM images of the bi-layer hydrogel. (**b**) The SEM images of the RFH and MPH. (**c**) Frequency sweep data of MPH in terms of G′ and G″ at 20 °C and 40 °C.

The basic properties of the RFH/MPH bi-layer hydrogels were measured (Figure 2). Scanning electron microscope (SEM) images of the bi-layer hydrogel manifested a significant bi-layer structure with a good bonding interface between the RFH layer and the MPH layer (Figure 2a). Compared with the SEM images of single MPH and RFH (Figure 2b), the different SEM images of the interface further confirmed the interface between the two hydrogel layers. In addition, the EDS mapping of the bi-layer hydrogel cross-section further confirmed the distribution of the C, N, and O elements in the hydrogel (Figure S2). Furthermore, the mechanical properties of the bi-layer hydrogel were tested. The bi-layer hydrogel was prepared by the interpenetration of two crosslinked networks of RFH and MPH, which made the interface with a relatively dense interpenetrating polymeric network structure between the bi-layers hydrogels. This kind of interface can increase the tensile strength of the bi-layer hydrogel but decrease its breaking elongation, which can also firmly bond for various complex shape changes without exfoliation (Figure S3). Although the breaking elongation of the bi-layer hydrogel was lower than that of a single MPH hydrogel or RFH, its two hydrogel layers can remain together for various complex shape changes. The addition of melanin effectively improved the mechanical properties of the hydrogel, which can be proved by the rheological experiment shown Figure S4. It was observed that the storage modulus (G′) and loss modulus (G″) of MPH were significantly higher than that of the PNIPAM hydrogel in the entire frequency range (0.1–100 rad/s) at 20 °C. In addition, rheological experiments at 20 °C and 40 °C showed that when the MPH was at 40 °C, G′ and G″ were significantly lower than those at 20 °C (Figure 2c), which indicated that the temperature of the external environment can influence the deforming performance of the hydrogel.

### 2.2. pH-Responsive Fluorescent Color-Changing Performance of the RFH LAYER

**Figure 3:**
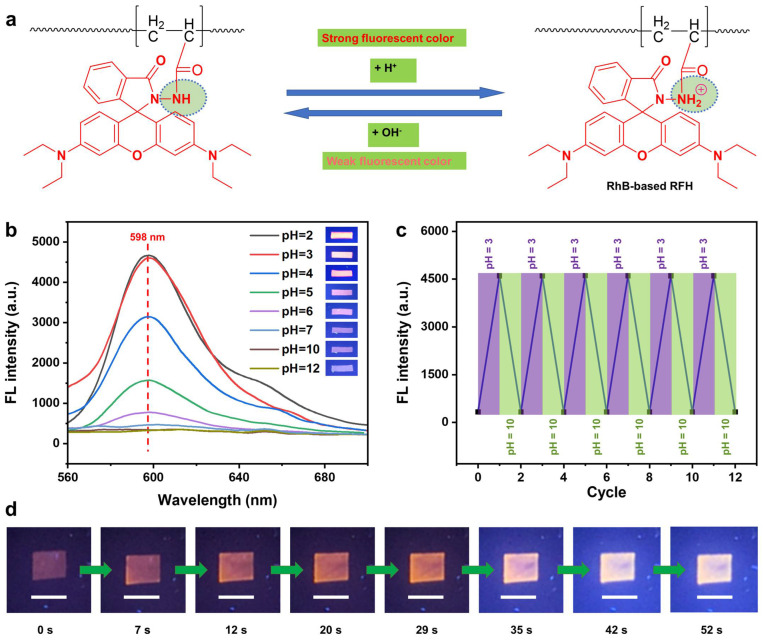
(**a**) The mechanism of pH-responsive fluorescent color-changing performance of the RFH. (**b**) The pH-dependent fluorescent emission spectra of RFH (λex = 365 nm). (**c**) The six cycles between pH = 3 and 10 buffer solution of the RFH. (**d**) The color-changing process of the RFH from pH = 10 to 3.

The pH-responsive fluorescent color-changing performance of RFH was researched (Figure 3). The mechanism of the pH responsiveness of RFH is illustrated in Figure 3a. As the fluorescent luminescence type of the RhB group belongs to “aggregation caused quenching” (ACQ), when the pH was changed from 10 to 3, the neutral imino of the RhB group transformed into a cation with a positive charge, which reduced the concentration of RhB groups due to the mutual repulsion of cations. Furthermore, the fluorescent color intensity of RFH was detected. As shown in Figure 3b, under ultraviolet (UV) light (365 nm), the immediate pH-responsive fluorescent color changes of RFH range from almost colorless transparent to orange. When the bi-layer hydrogel was immersed in different solutions with pH values ranging from 10 to 3, the fluorescent intensity at 598 nm was enhanced more than 10 times. The fluorescent response of RFH was completely reversible (Figure 3c). In addition, when the RFH was transferred from pH = 10 to pH = 3, the entire discoloration process was recorded (Figure 3d, Movie S1); it turned orange in a very short time and gradually became the brightest at 52 s. Therefore, these results indicate that RFH exhibited an excellent pH-responsive fluorescent color-changing performance.

### 2.3. Photothermal-Responsive Shape-Changing Performance of the MPH Layer

**Figure 4:**
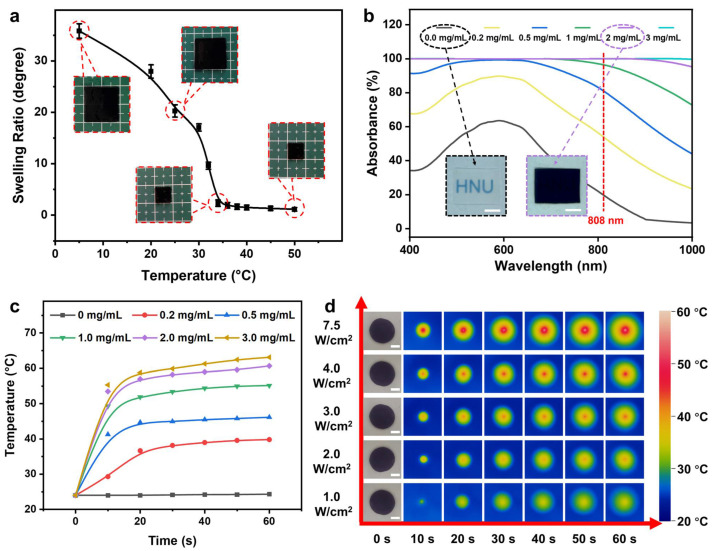
(**a**) Swelling ratio curve of MPH (2 mg/mL). (**b**) Absorbance of different concentrations of melanin solutions. (**c**) Temperature elevation curves of MPH with different melanin concentrations exposed to 808 nm NIR light (7.5 W/cm^2^). (**d**) IR thermal images of MPH (2.0 mg/mL) irradiated by different NIR light power. All scale bars are 5 mm.

The photothermal responsive shape-changing performance of the MPH layer was also investigated (Figure 4). The volume of the PNIPAM hydrogel shrank with an increase in temperature, and the addition of melanin made the PNIPAM hydrogel have an excellent photothermal response. Figure S5 shows the thermogravimetry (TG) and derivative thermogravimetry (DTG) curves of the dried PNIPAM hydrogel/MPH. It can be seen that dried PNIPAM hydrogel and MPH both have significant weightlessness steps at the range of 310–440 °C, indicating the complete pyrolysis of polymeric gel. Furthermore, compared to the PNIPAM gel, the decomposition temperature of the MPH changed from 408 °C to 395 °C, which indicated that the composited melanin can only slightly decrease the thermostability of the PNIPAM gel. Figure S6 shows the DSC thermal analysis curves of the PNIPAM hydrogel and MPH. The phase transition peaks of PNIPAM hydrogel and MPH were 32.5 °C and 33.4 °C, respectively, corresponding to the lower critical solution temperature (LCST) value of the hydrogel. It can be seen that the incorporation of melanin can slightly increase the LCST of the hydrogel, which may be due to a large number of -OH groups of melanin that can interact with NH2 groups of the PNIPAM chain segment to form hydrogen bonds.

The swelling ratio of MPH was tested. As shown in Figure 4a, the volume of MPH swelled slightly from a temperature of 25 °C to cold water at 5 °C. With the continuous increase in temperature, the volume shrank sharply at 32 °C and then tended to be flat. Based on the volume change, a greater actuating force can be provided to the hydrogel actuator. In order to quantitatively study the amount of melanin and make MPH have better photothermal conversion performance, the proportion screening of melanin concentration was carried out. Firstly, the concentrations of melanin of 0.2 mg/mL, 0.5 mg/mL, 1 mg/mL, 2 mg/mL, and 3 mg/mL were selected (Figure S7), and thermal images tests were conducted on the different concentrations of melanin (Figures S8 and S9). It can be seen that a small amount of melanin under NIR light irradiation rose to higher temperatures in a very short time. Secondly, MPH with different concentrations of melanin was tested using an ultraviolet–visible (UV–Vis) absorption spectra spectrophotometer (Figure 4b). It can be seen that MPH had better light absorption capacity and photothermal performance with increasing melanin concentration. MPH with melanin concentrations of 2 mg/mL and 3 mg/mL showed similar light absorption at 808 nm. So, 2 mg/mL MPH of the bi-layer hydrogel can achieve fast actuating performance, with no need for a higher concentration.

Figure 4c shows the temperature elevation curves of MPH with different concentrations of melanin under near-infrared light at 808 nm and IR thermal images, as shown in Figure S10. It can be seen that the rising curves of the 2 mg/mL and 3 mg/mL concentrations of MPH were very close. So, combined with the light absorption capacity of MPH, 2 mg/mL of melanin was selected to prepare the hydrogel. Secondly, we studied the temperature rise curve of MPH (2 mg/mL) with time under different light intensities, as shown in Figure S11 and the IR thermal images (Figure 4d). The addition of melanin was an important prerequisite for the photothermal deformation of bi-layer hydrogels. It was very important that melanin does not leak out of the hydrogel network, even during the swelling and actuation processes. We conducted 20 cycles of swelling and 20 cycles of actuating behavior of the MPH hydrogel and performed UV–Vis absorption spectra tests before and after actuating (Figure S12). It can be seen that the transmittance of the MPH hydrogel remained unchanged during multiple swelling and actuation processes, showing good stability. The immobilization of the melanin micromolecules in the hydrogel network without leakage is probably due to the hydrogen bonds between the hydroxyl groups of melanin and acylamino groups of the PNIPAM.

### 2.4. NIR-Responsive Shape-Changing Performance of the Bi-Layer Hydrogel

**Figure 5:**
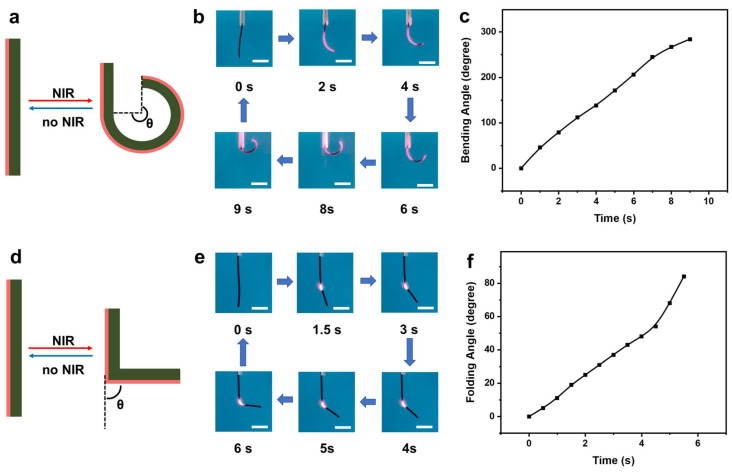
(**a**–**c**) The bending process of the bi-layer hydrogel actuator under 7.5 W/cm^2^ of NIR light irradiation. (**d**–**f**) The folding process of the bi-layer hydrogel actuator under 60 W/cm^2^ of NIR light irradiation. All scale bars are 5 mm.

The volume of the MPH shrank with the increase in temperature, and the other layer of the RFH limited the shrinkage of MPH, which resulted in internal stress and deformations of the actuator. Based on this property, the specific behavior (bending and folding) of the composite hydrogel actuator under near-infrared irradiation was quantitatively analyzed (Figure 5). First of all, under the area irradiation of NIR light, MPH was heated, the volume of the MPH layer was reduced, and the volume of the RFH layer was unchanged; thus, the bi-layer hydrogel generated internal stress and produced bending behavior (Figure 5a). Next, the composite hydrogel actuator was cut into strips of 10 × 2 × 0.4 mm^3^, and then the bending and folding behavior of the bi-layer hydrogel was studied. Under the area irradiation of NIR light (with a 5.2 W/cm^2^ intensity of an 808 nm laser lamp), the LCST of MPH can be reached in a very short time because of the excellent photothermal conversion performance of MPH and then shrank, resulting in the bending behavior of the bi-layer hydrogel; the composite hydrogel actuator can bend to 284° within 9 s (Figure 5b,c).

Next, we investigated the folding behavior of the bi-layer hydrogel actuation. Under NIR point light irradiation, the MPH layer was heated locally, resulting in volume shrinkage. The volume shrinkage was restricted by the RFH hydrogel, resulting in folding behavior (Figure 5d). Under the point irradiation of NIR light (with 60 W/cm^2^ intensity of an 808 nm laser lamp), the bi-layer hydrogel actuator can fold to 90° within 6 s (Figure 5e,f), with a faster response and recovery speed compared to the bending behavior (Figure S13).

### 2.5. The Bi-Functional Synergy Process of the Bi-Layer Hydrogel in the Dark

**Figure 6:**
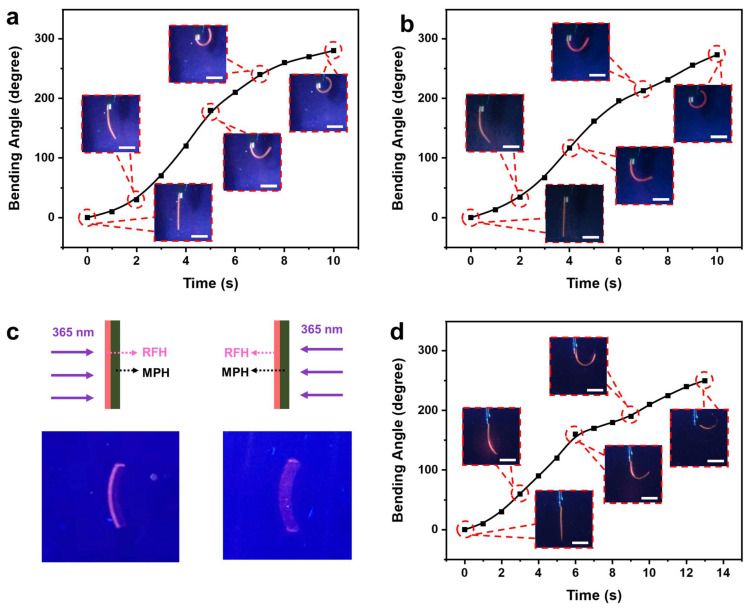
(**a**) The bending process of the bi-layer hydrogel actuator under 7.5 W/cm^2^ of NIR light irradiation at 20 °C and pH = 3 in the dark. (**b**) The bending process of the bi-layer hydrogel actuator under 7.5 W/cm^2^ of NIR light irradiation at 20 °C and pH from 10 to 3 in the dark. (**c**) The illustration of bi-layer hydrogel fluorescence intensity in different directions under UV light irradiation. (**d**) The bending process of the bi-layer hydrogel actuator under 7.5 W/cm^2^ of NIR light irradiation with single-direction UV light irradiation. All scale bars are 5 mm.

The bi-layer hydrogels showed fluorescent orange under acidic conditions under 365 nm UV light, and the shape change can be achieved by NIR light irradiation. Under the synergistic effect of these bi-functions, the bi-layer hydrogel actuator can realize a precisely programmed shape change, which can be synchronously tracked under dark conditions (Figure 6, Figures S12 and S13). Firstly, the hydrogel actuator was cut into long strips of 10 × 2 × 0.4 mm^3^ and placed in an acidic solution (pH = 3) at 20 °C. Under UV light, the hydrogel actuator exhibited pH-responsive fluorescence. At the same time, under the area irradiation of NIR light (5.2 W/cm^2^ intensity of an 808 nm laser lamp), the long-strip hydrogel actuator can achieve a bending process (Figure 6a), and the bending process can be located by the fluorescence emitted by the RFH layer. Under this shape-changing/color-changing bi-functional synergy, the bi-layer hydrogel can provide a bending process in the dark. Similarly, under the irradiation of NIR point light, the bi-layer hydrogel can achieve folding behavior due to the synergistic effect of bi-function, and the folding behavior can also be displayed in the dark (Figure S14).

In addition to tracking its deformation in the dark under acidic and NIR light irradiation, this bi-functional synergistic effect can also synchronously realize fluorescent color and shape changes (Figure 6b). The hydrogel actuator was cut into long strips of 10 × 2 × 0.4 mm^3^ and placed in an alkaline solution at 20 °C with pH = 10. Hydrogel actuators have almost no fluorescent effect under UV light. Under the area irradiation of NIR light (5.2 W/cm^2^ intensity of an 808 nm laser lamp), the strip of the hydrogel actuator began to bend. At the same time, acid was added to the solution to make it acidic. With the bending process of the bi-layer hydrogel actuator, the RFH layer also began to synchronously fluoresce and became increasingly brighter with increasing acidity. Consequently, the bending behavior of the bi-layer hydrogel was gradually shown in the dark.

MPH can absorb most of the light, including the orange fluorescence emitted by the RFH layer; so, MPH can also blocked-out fluorescent (Figure 6c). RFH can be fluoresced when exposed to 365 nm UV light, and the MPH was oriented towards the UV light source; RFH only emitted a faint fluorescent effect because most of the light was absorbed. Under 7.5 W/cm^2^ of NIR light irradiation with single-direction UV light irradiation, the bi-layer hydrogel can bend, and the bi-layer hydrogel gradually moves away from the direction of UV light (Figure 6d). Meanwhile, the RFH layer was shielded by the MPH layer, and the fluorescent effect gradually vanished, allowing real-time tracking of the process.

### 2.6. Biomimetic Devices Based on the Bi-Layer Hydrogel with Bi-Functional Synergy

**Figure 7:**
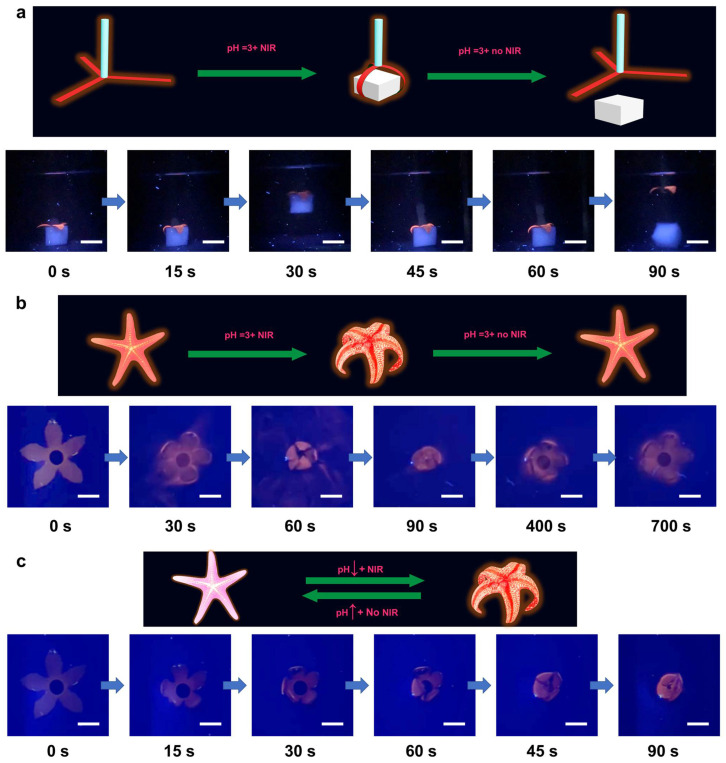
(**a**) The tracing process of the biomimetic “gripper” in the dark. (**b**) The tracing process of the biomimetic “starfish” in the dark. (**c**) The synchronous color-changing and shape-changing process of biomimetic “starfish” in the dark. All scale bars are 5 mm.

Based on the NIR-responsive complex shape change and the pH-responsive fluorescent color change of this bi-layer hydrogel, several biomimetic devices were designed (Figure 7). Under dark conditions, common stimuli-responsive hydrogels cannot be observed. However, this bi-layer hydrogel can be designed as a biomimetic “gripper”, which can show fluorescent color for real-time tracking in the dark in an aqueous solution at pH = 3 (Figure 7a, Movie S2). Upon NIR light irradiation, the MPH was heated and the bi-layer hydrogel bent immediately; thus, the biomimetic “gripper” grabbed the object tightly and then released in a specific location without NIR light irradiation. That is, all the “grabbing” and releasing” processes can be clearly tracked in the dark.

Furthermore, a biomimetic “starfish” was designed based on this bi-layer hydrogel (Figure 7b,c). Similarly to the biomimetic “gripper”, the shape-changing process of this biomimetic “starfish” can also be easily traced in the dark (Figure 7b, Movie S3). When the biomimetic “starfish” was placed in an acidic solution with pH = 3, the fluorescence of the RFH layer was obvious, but because of the blocked MPH layer, the bi-layer hydrogel was not obvious. Under the area irradiation of NIR light (5.2 W/cm^2^ intensity of an 808 nm laser lamp), the biomimetic “starfish” began to move, and the actuation behavior was recorded in the dark. As the “tentacles” of the biomimetic “starfish” gradually curved, the biomimetic “starfish” showed bright fluorescence. When the NIR light was removed, the bionic starfish gradually returned to their initial state.

Most importantly, similarly to natural starfish, this biomimetic “starfish” can simultaneously change its color and shape (Figure 7c, Movie S4) by synchronously NIR-irradiating it and changing the pH from 10 to 3; this “starfish” curled its body and showed increasingly strong fluorescent orange color at the same time. That is, this biomimetic “starfish” can provide color-changing and shape-changing bi-functional synergy.

## 3. Conclusions

In summary, we have explored an anisotropic bi-layer hydrogel by combining a pH-responsive rhodamine B (RhB)-functionalized hydrogel layer and a photothermal-responsive shape-changing poly (N-isopropylacrylamide) (PNIPAM) hydrogel layer with fluorescent color-changing and shape-changing bi-functional synergy. This bi-layer hydrogel can obtain fast and complex actuations under irradiation with 808 nm near-infrared (NIR) light due to both the melanin-composited PNIPAM hydrogel with high efficiency of photothermal conversion and the anisotropic structure of this bi-hydrogel. Furthermore, the RhB-functionalized fluorescent hydrogel layer can provide rapidly pH-responsive fluorescent color change, which can be integrated with NIR-responsive shape change to achieve bi-functional synergy. As a result, this bi-layer hydrogel can be designed using various biomimetic devices, which can show the actuating process in the dark for real-time tracking and even mimetic starfish synchronously change both the color and shape. This work provides a new bi-layer hydrogel biomimetic actuator with color-changing and shape-changing bi-functional synergy, which will inspire new strategies for other intelligent composite materials and high-level biomimetic devices.

## 4. Materials and Methods

### 4.1. Materials

Acrylamide (Am, 96%), N, N′-Methylenebis(acrylamide) (BIS, 99%), ammonium persulfate (APS), tetramethylethylenediamine (TEMED, 99%), ammonium peroxodisulfate (APS, 98%), Rhodamine B (RhB), acetonitrile (CH_3_CN), acryloyl chloride (C_3_H_3_ClO), dichloromethane (CH_2_Cl_2_), sodium bicarbonate (NaHCO_3_), and sodium sulfate (Na_2_SO_4_) were purchased from Macklin Inc., Shanghai, China. N-isopropylacrylamide (NIPAM, 98%) was purchased from Sigmar, Shanghai, China. Water-soluble melanin and polyvinyl alcohol-124 (PVA-124, Mw~195000) were purchased from Aladdin, Shanghai, China. All the chemical reagents were used as received.

### 4.2. Synthesis of pH-Responsive RhB

The pH-responsive RhB was polymerized in the following steps. Firstly, 5 g of rhodamine B and 8 mL of hydrazine hydrate were dissolved in 80 mL of methanol. The reaction mixture was stirred at 80 °C and refluxed for 24 h. The solvent was removed under decreased pressure, after which 200 mL of CH_2_Cl_2_ was added, and the solvent was washed with water several times. RhB hydrazide was obtained as a pink solid. The RhB hydrazide and dry CH_3_CN were mixed until the RhB hydrazide was completely dissolved. Then, 8 mL of C_3_H_3_ClO was added dropwise into the mixture, which was stirred under reflux at 60 °C for 2 h. The mixture was filtered and washed three times with dry CH_3_CN. The crude product was dissolved in 200 mL of CH_2_Cl_2_ and washed several times in a saturated NaHCO_3_ aqueous solution. Then, the organic phase was dried on anhydrous Na_2_SO_4_ and filtered. After all the solvents were removed, the final product was a violet solid, which was a pH-responsive RhB.

### 4.3. Bi-Layer Actuator Preparation

First, the MA-PNIPAM layer was polymerized under a 0.2 mm silicone rubber mold by mixing 100 mg of NIPAM, 5 mg of BIS, 2 mg of water-soluble melanin, 8 μL of TEMED, 80 μL of 4% APS solution, and 1 mL of 1% PVA solution. Then, the preformed MPH was immersed in deionized water for standby applications. Second, the MPH was placed on the glass, and double 0.2 mm silicone rubber molds were added around it. The RhM-Am precursor was added to the PNIPAM hydrogel and sealed with another glass. The Am precursor was prepared by mixing Am (100 mg), BIS (3 mg), pH-responsive RhB (4 mg), TEMED (4 μL), 4% of APS solution (40 μL), and 1 mL of deionized water.

### 4.4. Characterization of Actuator

The morphology characterization and elemental distribution of the blank hydrogel, RFH, MPH, and the actuator were observed using a scanning electron microscope (SEM, HITACHI S-4800, Hitachi High Technologies, Ibaraki Prefecture, Japan). The RhB was tested using a nuclear magnetic resonance spectrometer (Bruker AM 300, Bruker, MA, USA). The hydrogel with RhB was tested using a fluorospectrophotometer (F-4600, Hitachi, Tokyo, Japan). IR thermal images were obtained using an infrared thermal imaging instrument (Rx-500, Bufan, Beijing, China). The actuation was obtained using an infrared laser emitter(VCL-808, Lantu, Beijing, China). Ultraviolet–visible (UV–Vis) absorption spectra were obtained using a spectrophotometer (UV1800PC, AuCy Instrument, Aoyan, Shanghai, China). The mechanical properties of the RFH, MPH, and actuator were tested using a universal testing machine (GP-6113A, GAOPIN, Suzhou, Germany). The pH was measured and adjusted using a pH meter (OHAUS ST20, Parsippany, NJ, USA). The optical images and movies were photographed using an iPhone 13 Pro.

### 4.5. Swelling Ratio Test of Hydrogel

MPH was used to determine the equilibrium swelling ratio (*SR*) in deionized water at different temperatures. Firstly, the prepared MPH (50 × 50 × 0.3 mm^3^) and added to the deionized water for full swelling. Second, the hydrogel was placed in deionized water at different temperatures (5–50 °C) for at least 3 h. Therefore, the weights after full swelling at each of the water temperatures were recorded as *W_s_*, which needed wiping the surface water of the hydrogel. Finally, the freeze-dried hydrogel weight was recorded as *W_d_*. Additionally, the *SR* was calculated using the following Equation (1).

(1) $$SR\%=\frac{W_{s}-W_{d}}{W_{d}}\times 100\%$$

## Data Availability

The data in this work are available from the corresponding authors upon reasonable request.

## Supplementary Materials

The following supporting information can be downloaded at: https://www.mdpi.com/article/10.3390/gels9060438/s1, Figure S1: (a) Synthesis and preparation of RhB acrylamide. (b) HNMR of RhB hydrazide. (c) HNMR of RhB acrylamide; Figure S2: The EDS-mapping of bi-layer hydrogel; Figure S3: The stress–strain curve of MPH, RFH, and bi-layer hydrogel; Figure S4: Storage modulus (G′) and Loss modulus (G″) of MPH and PNIPAM hydrogel at 25 °C; Figure S5: TG and DTG curve of (a) PNIPAM hydrogel and (b) MPH; Figure S6: DSC curve of PNIPAM hydrogel and MPH; Figure S7: Different concentrations of melanin and blank control group; Figure S8: Thermal images of different concentrations of melanin; Figure S9: Curves of different concentrations of melanin over time; Figure S10: Temperature rising curve of MPH under different near-infrared light intensities; Figure S11: Thermal imaging of MPH under different near-infrared light intensities; Figure S12: Absorbance of MPH (2 mg/mL) in actuating before (a) and after (b); Figure S13: Folding recovery process; Figure S14: Deformation in water at 40 °C; Figure S15: In the dark, deformation in water at 40 °C and pH = 3; Figure S16: The folding process of the bi-layer hydrogel actuator under 60 W/cm^2^ of NIR light irradiation at 20 °C and pH = 3 in the dark. Movie S1. The fluorescent color-changing kinetics of the RFH layer. Movie S2. The tracing process of the biomimetic gripper in the dark. Movie S3. The tracing process of the biomimetic “starfish” in the dark. Movie S4. The synchronous shape-changing and color-changing process.
