# Supplementary material for: Anisotropic Bi-Layer Hydrogel Actuator with pH-Responsive Color-Changing and Photothermal-Responsive Shape-Changing Bi-Functional Synergy
Source: Gels. 2023 May 25;9(6):438. doi: 10.3390/gels9060438 (PMC10297277; doi:10.3390/gels9060438)
Supplement: Supplementary file 1 [file gels-09-00438-s001.zip › gels-2402275 - Supporting information #1.pdf]

gels

MDPI

Supporting information

Anisotropic Bi-layer Hydrogel Actuator with pH-Responsive Color-Changing and Photothermal-Responsive Shape-Changing Bi-Functional Synergy

Chao Ma [1], Shuyi Peng [1], Lian Chen [2], Xingyu Cao [1,3], Ye Sun [1,3], Lin Chen [1], Lang Yang [1], Chunming Ma [3,*], Qijie Liu [4], Zhenzhong Liu [4], and Shaohua Jiang [2,*]

[1] State Key Laboratory of Marine Resource Utilization in South China Sea, Hainan University, Haikou 570228, China
[2] Jiangsu Co-Innovation Center of Efficient Processing and Utilization of Forest Resources, International Innovation Center for Forest Chemicals and Materials, College of Materials Science and Engineering, Nanjing Forestry University, Nanjing 210037, China
[3] Shenzhen Institute of Advanced Electronic Materials - Shenzhen Fundamental Research Institutions, Shenzhen Institutes of Advanced Technology, Chinese Academy of Sciences, Shenzhen 518055, China
[4] Taizhou Key Laboratory of Medical Devices and Advanced Materials, Research Institute of Zhejiang University-Taizhou, Taizhou 318000, China
* Correspondence: cm.ma@siat.ac.cn (C.M.); shaohua.jiang@njfu.edu.cn (S.J.)

Supporting Figures

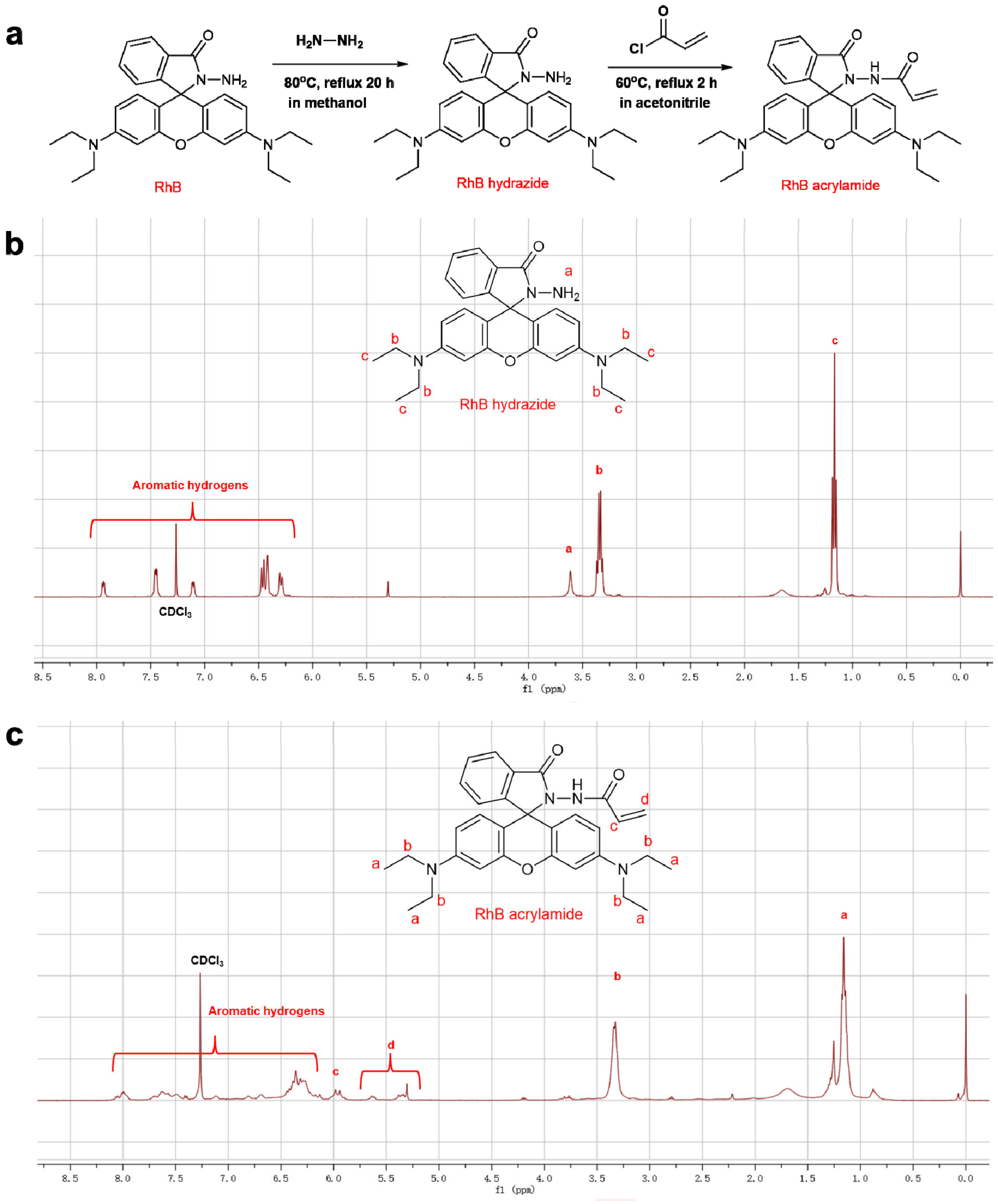

Figure S1. (a) Synthesis and preparation of RhB acrylamide. (b) HNMR of RhB hydrazide. (c) HNMR of RhB acrylamide.

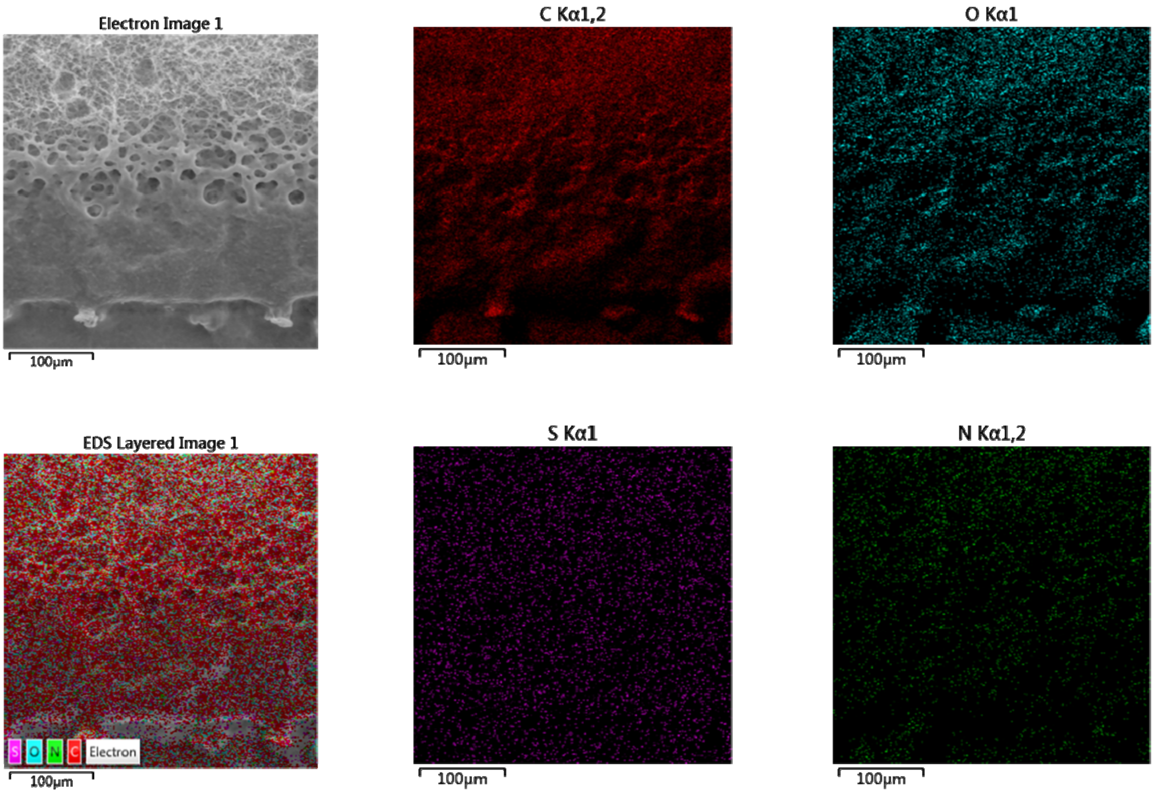

Figure S2. The EDS-mapping of bi-layer hydrogel.

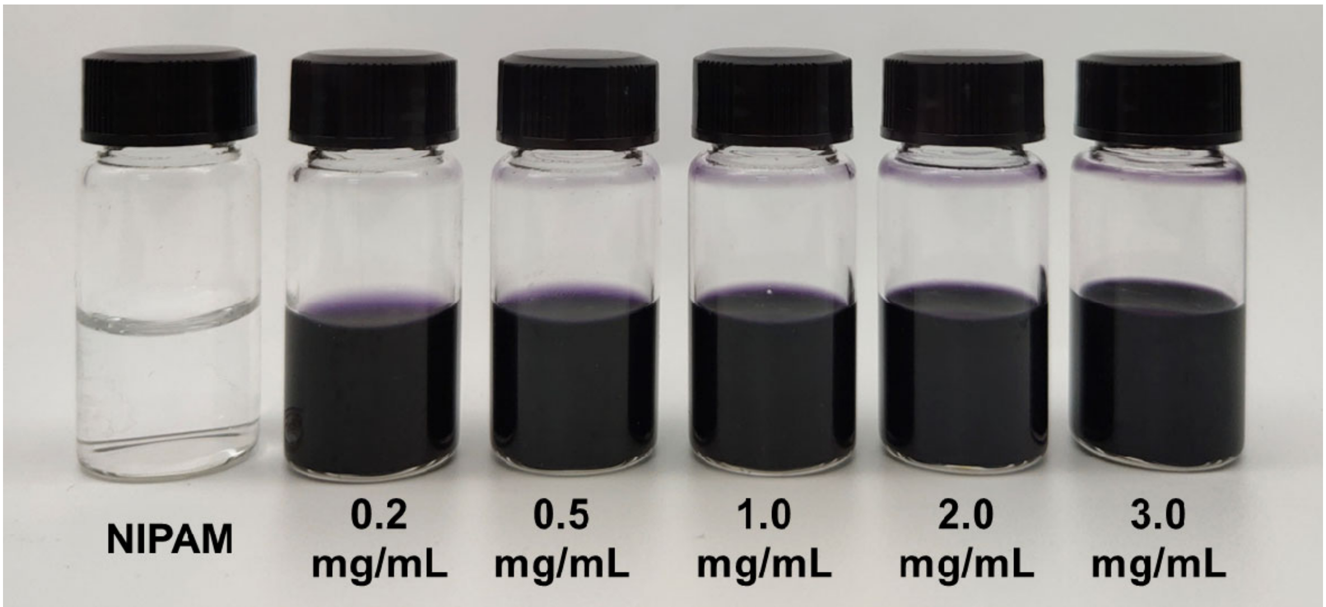

Figure S3. Different concentrations of melanin and blank control group.

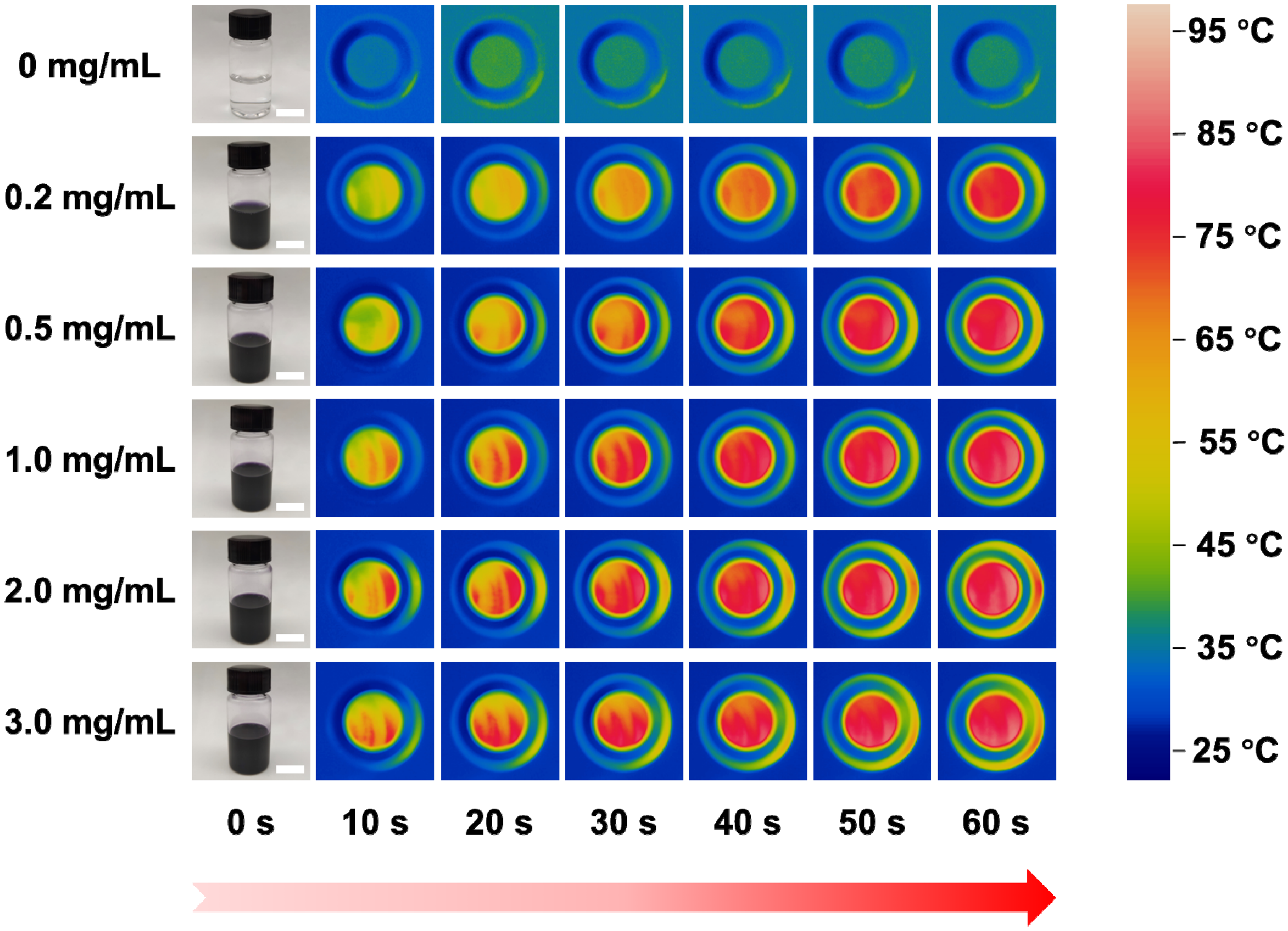

Figure S4. Thermal images of different concentrations of melanin.

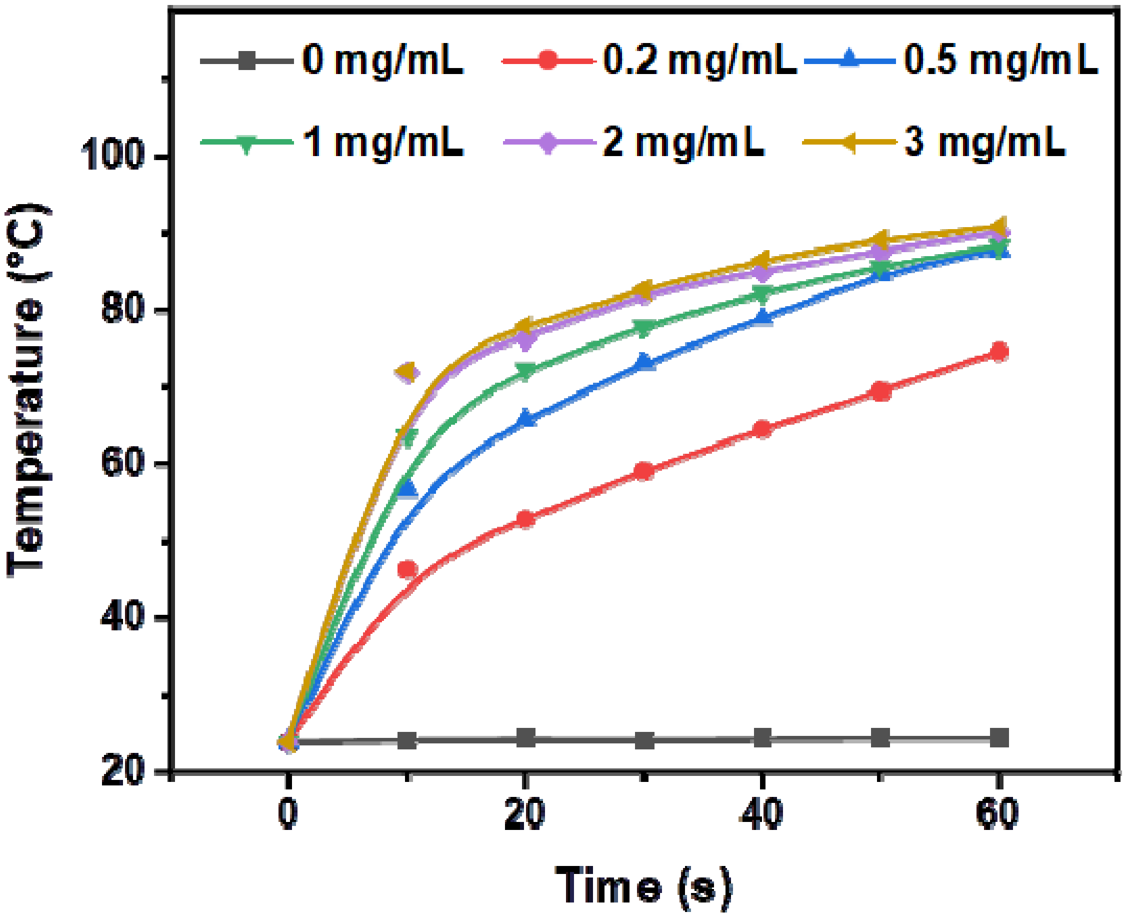

Figure S5. Curves of different concentrations of melanin over time.

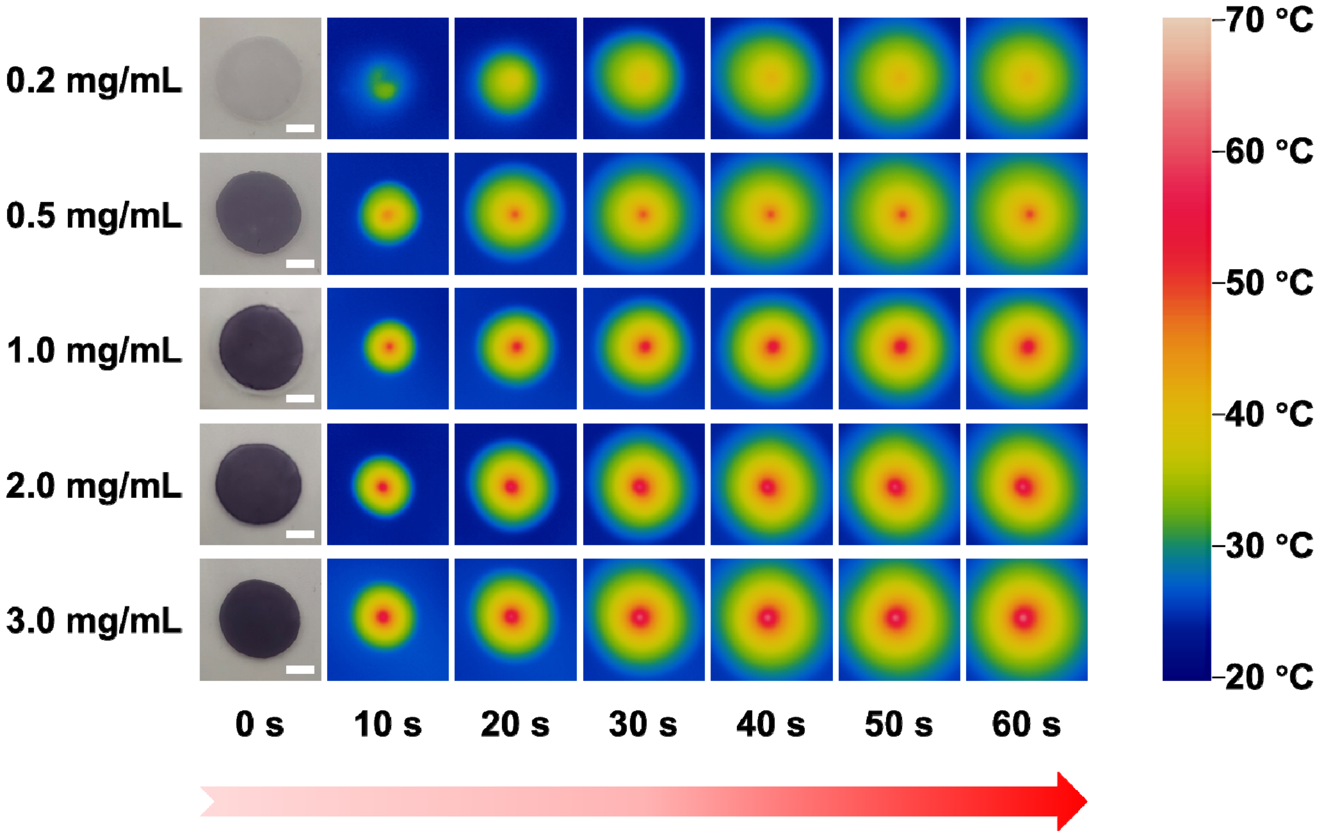

Figure S6. Temperature rise curve of MPH under different near infrared light intensities.

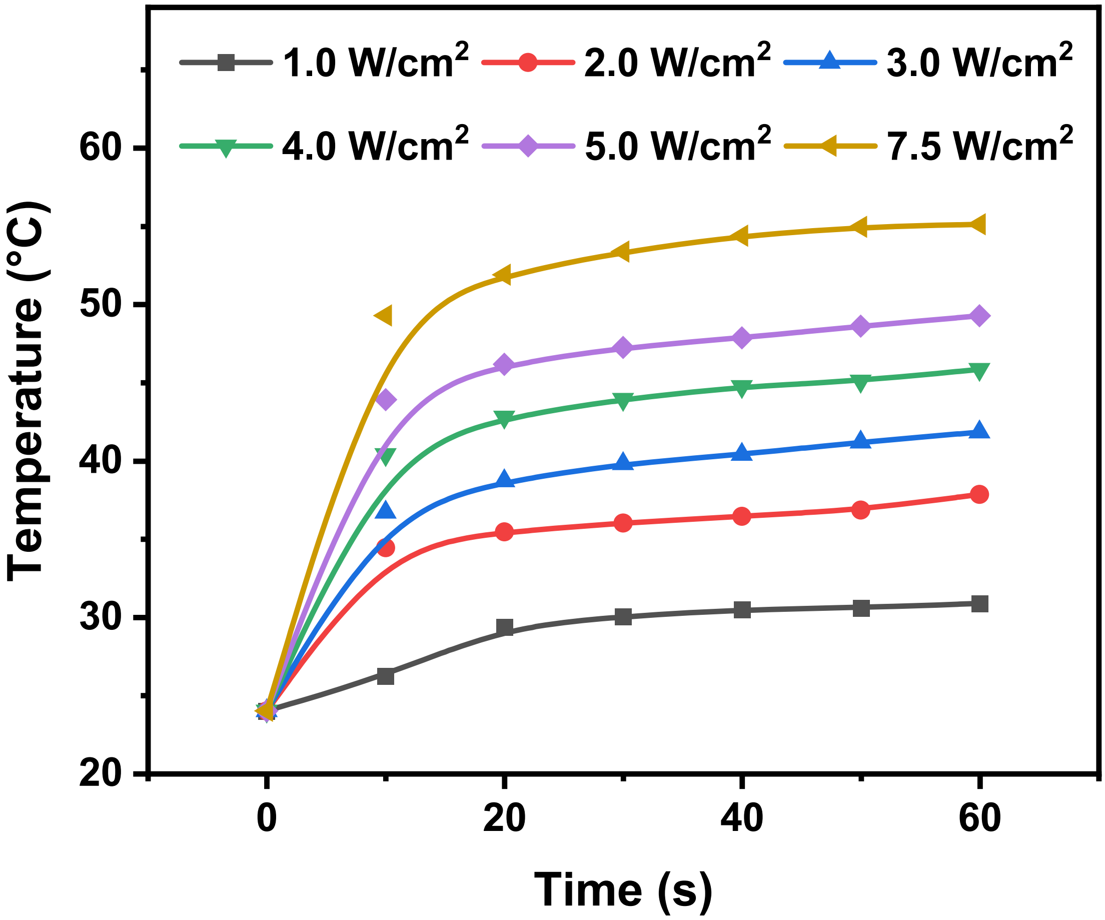

Figure S7. Thermal imaging of MPH under different near infrared light intensities.

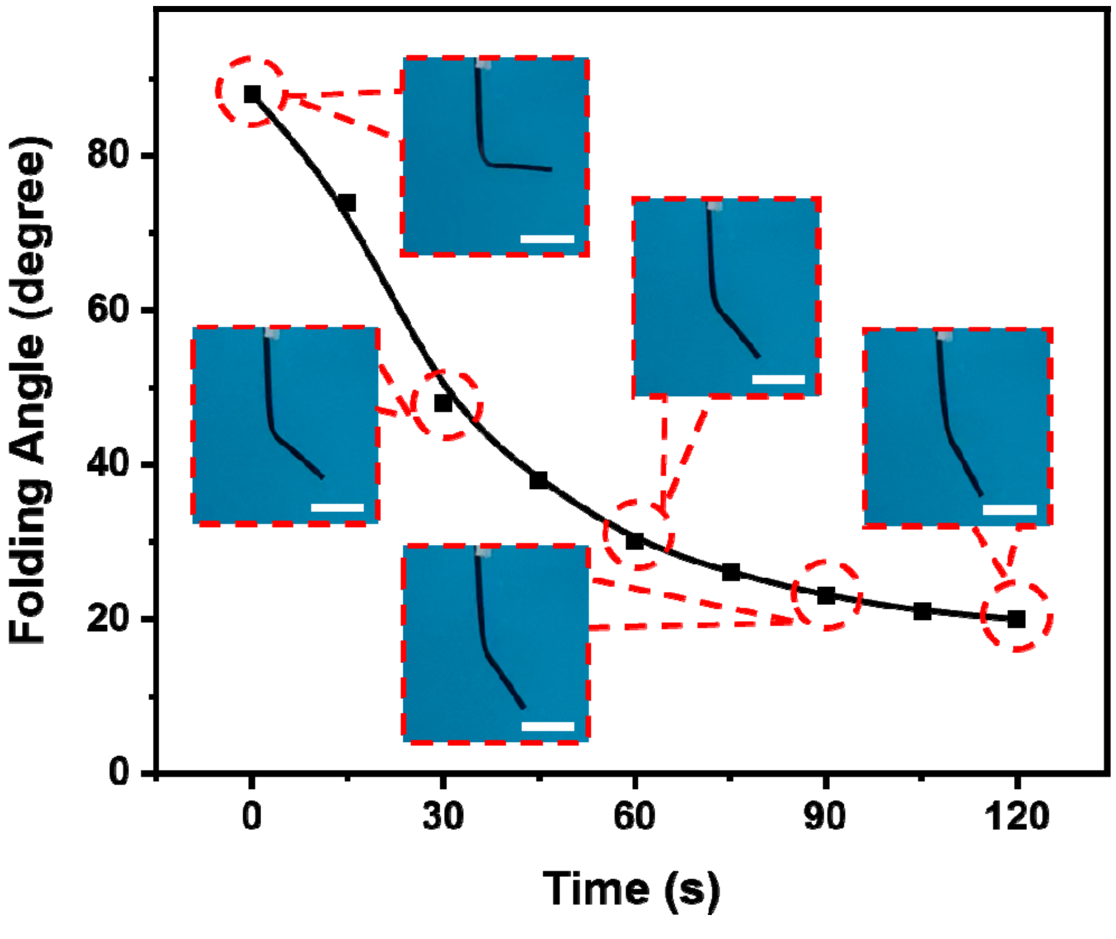

Figure S8. Folding recovery process.

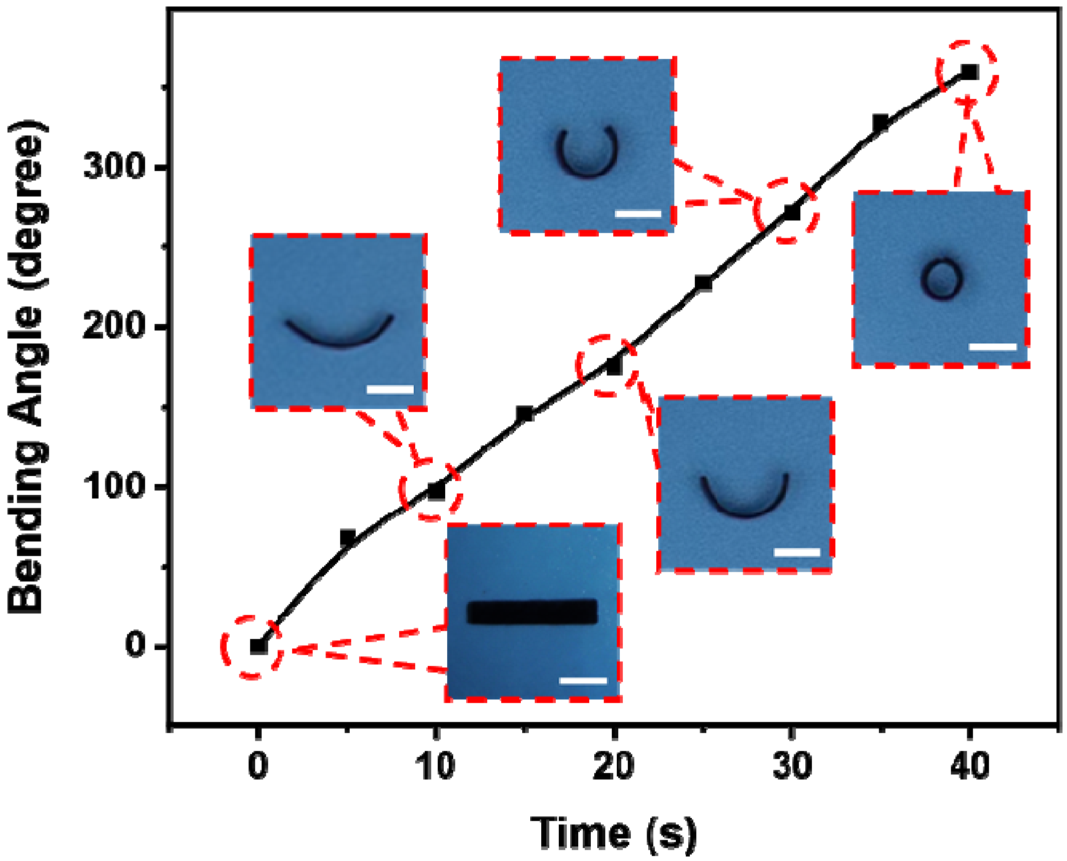

Figure S9. Deformation in water at 40℃.

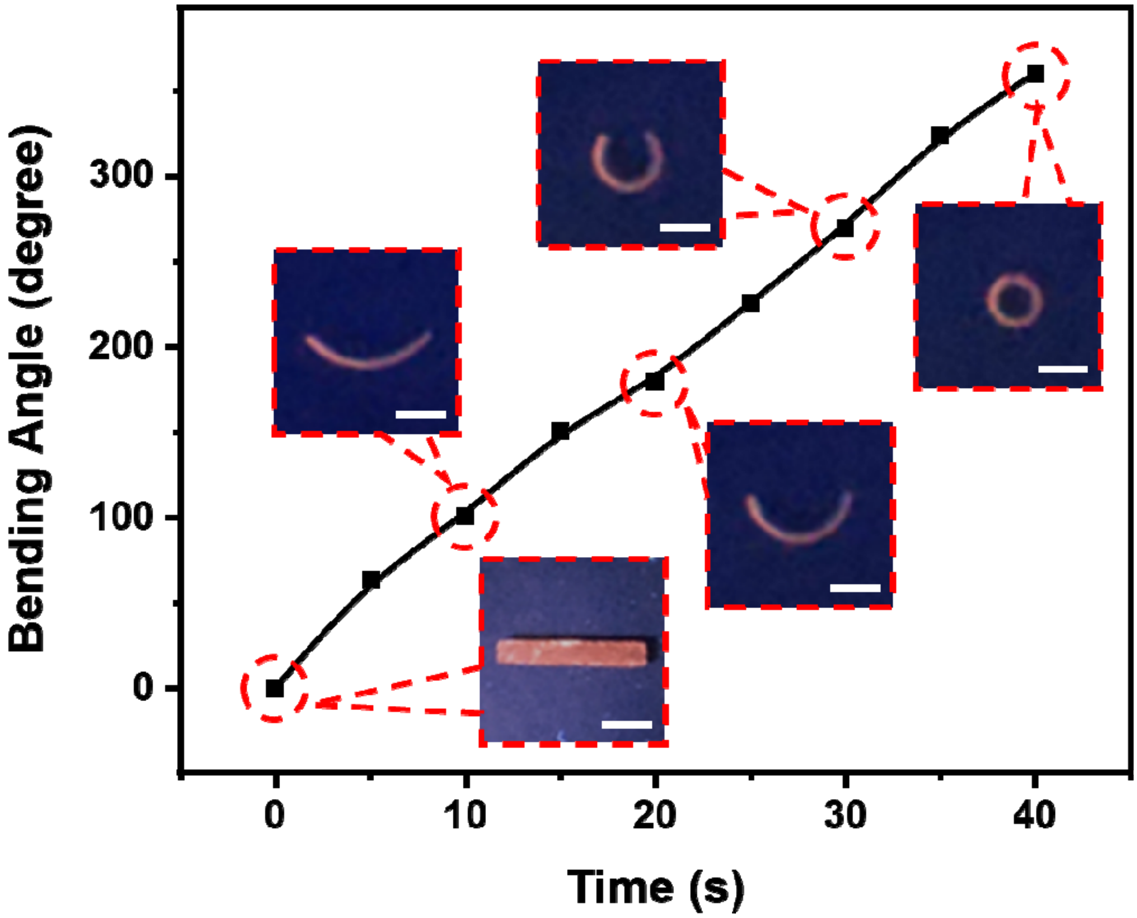

Figure S10. In the dark，deformation in water at 40℃.

Supporting Movies

Movie S1. The fluorescent color-changing kinetics of the RFH layer.

Movie S2. The tracing process of the biomimetic gripper in the dark.

Movie S3. The tracing process of the biomimetic "starfish" in the dark.

Movie S4. The synchronous shape-changing and color-changing process.
